# Pirfenidone attenuates lung fibrotic fibroblast responses to transforming growth factor-β1

**DOI:** 10.1186/s12931-019-1093-z

**Published:** 2019-06-11

**Authors:** Jin Jin, Shinsaku Togo, Kotaro Kadoya, Miniwan Tulafu, Yukiko Namba, Moe Iwai, Junko Watanabe, Kumi Nagahama, Takahiro Okabe, Moulid Hidayat, Yuzo Kodama, Hideya Kitamura, Takashi Ogura, Norikazu Kitamura, Kazuho Ikeo, Shinichi Sasaki, Shigeru Tominaga, Kazuhisa Takahashi

**Affiliations:** 1 Department of Respiratory and Critical Care Medicine, Beijing Hospital, National Center of Gerontology, Beijing, 100730 People’s Republic of China; 2 Division of Respiratory Medicine, Juntendo University Faculty of Medicine and Graduate School of Medicine, 2-1-1 Hongo, Bunkyo-ku, Tokyo, 113-8421 Japan; 3 Research Institute for Diseases of Old Ages, Juntendo University Graduate School of Medicine, 2-1 -1 Hongo, Bunkyo-ku, Tokyo, 113-8421 Japan; 4 Department of Respiratory Medicine Kanagawa Cardiovascular and Respiratory Center, 6-16-1 Tomiokahigashi, Kanazawa-ku, Yokohama, Kanagawa 236-0051 Japan; 5 Center for Information Biology, National Institute of Genetics, 1111 Yata, Mishima, Shizuoka, 411-8540 Japan; 6 Department of Genetics, SOKENDAI, 1111 Yata, Mishima, Shizuoka, 411-8540 Japan; 7 Department of Respiratory Medicine, Juntendo University Urayasu Hospital, Chiba, 279-0001 Japan; 8 Division of Respiratory Medicine, Juntendo University Faculty of Medicine and Graduate School of Medicine, 3-1-3 Hongo, Bunkyo-ku, Tokyo, 113-8421 Japan

**Keywords:** Pirfenidone, Collagen triple helix repeat containing protein 1(CTHRC1), Four-and-a-half LIM domain protein 2(FHL-2), BMP-4, Transforming growth factor-β1, Lung fibroblast, Lung fibrosis

## Abstract

**Background:**

Pirfenidone, an antifibrotic agent used for the treatment of idiopathic pulmonary fibrosis (IPF), functions by inhibiting myofibroblast differentiation, which is involved in transforming growth factor (TGF)-β1-induced IPF pathogenesis. However, unlike normal lung fibroblasts, the relationship between pirfenidone responses of TGF-β1-induced human fibrotic lung fibroblasts and lung fibrosis has not been elucidated.

**Methods:**

The effects of pirfenidone were evaluated in lung fibroblasts isolated from fibrotic human lung tissues after TGF-β1 exposure. The ability of two new pharmacological targets of pirfenidone, collagen triple helix repeat containing protein 1(CTHRC1) and four-and-a-half LIM domain protein 2 (FHL2), to mediate contraction of collagen gels and migration toward fibronectin were assessed in vitro.

**Results:**

Compared to control lung fibroblasts, pirfenidone significantly restored TGF-β1-stimulated fibroblast-mediated collagen gel contraction, migration, and CTHRC1 release in lung fibrotic fibroblasts. Furthermore, pirfenidone attenuated TGF-β1- and CTHRC1-induced fibroblast activity, upregulation of bone morphogenic protein-4(BMP-4)/Gremlin1, and downregulation of α-smooth muscle actin, fibronectin, and FHL2, similar to that observed post-CTHRC1 inhibition. In contrast, FHL2 inhibition suppressed migration and fibronectin expression, but did not downregulate CTHRC1.

**Conclusions:**

Overall, pirfenidone suppressed fibrotic fibroblast-mediated fibrotic processes via inverse regulation of CTHRC1-induced lung fibroblast activity. Thus, CTHRC1 can be used for predicting pirfenidone response and developing new therapeutic targets for lung fibrosis.

**Electronic supplementary material:**

The online version of this article (10.1186/s12931-019-1093-z) contains supplementary material, which is available to authorized users.

## Background

Accumulation of activated lung myofibroblasts and excessive deposition of extracellular matrix (ECM) produced by these cells result in lung tissue contraction, as has been observed in fibrotic lung tissues [1]. This can disrupt lung function, and therefore, inhibition of fibrotic processes may alter the progression of lung fibrosis-related diseases. Diverse mediators, including Krebs von den Lungen (KL)-6 and surfactant protein (SP)-D released from damaged epithelial cells, and inflammatory cytokines [2, 3] and pro-fibrotic growth factors (e.g., transforming growth factor [TGF]-β1 and platelet-derived growth factor [PDGF]) secreted by infiltrated inflammatory cells during airway inflammation induce fibrosis via autocrine mechanisms, which involves local lung fibroblast activation in the interstitial alveolar septa [4, 5].

TGF-β1, a key mediator of normal tissue repair [6], strongly stimulates mesenchymal cells to produce large amounts of ECM, including fibronectin and collagen, resulting in the development of fibrosis [4]. In addition, TGF-β1 stimulates fibroblast chemotaxis toward fibronectin [7, 8] and augments fibroblast-mediated contraction of ECM by stimulating contractile stress fibers (α-smooth muscle actin [α-SMA]) [9], generating lung fibroblasts that can be used as in vitro model of lung fibrosis. Fibronectin released from lung fibroblasts is a known autocrine or paracrine mediator of lung fibroblast-dependent chemotaxis and collagen gel contraction [8, 10]. We demonstrated that fibrotic fibroblasts, characterized by high response to TGF-β1, stimulated TGF-β1-induced contraction of collagen gels, fibroblast migration, and expression of α-smooth muscle actin and fibronectin. The ERK5 inhibitor blocked these responses more in fibrotic fibroblasts than in normal lung fibroblasts [11]. These observations suggest that fibrotic fibroblasts respond specifically to fibroblast-mediated fibrotic processes and anti-fibrotic compounds.

Previous reports have shown that corticosteroid treatment does not improve prognosis in patients with idiopathic pulmonary fibrosis (IPF) [12], suggesting that antifibrotic agents may be more useful than anti-inflammatory agents for the treatment of IPF. Pirfenidone (5-methyl-1-phenyl-2-(1H)-pyridone) is a potent antifibrotic agent that can inhibit the progression of fibrosis in patients with IPF. Pirfenidone attenuates transcription of procollagen, TGF-β1, and PDGF and ameliorates bleomycin-induced lung fibrosis in a rodent model [13–15]. However, the precise mechanisms via which pirfenidone suppresses lung fibrosis are still unclear.

In this study, we evaluated the effects pirfenidone on TGF-β1-mediated contraction of ECM and migration of lung fibroblasts isolated from patients with lung fibrosis toward fibronectin and compared them with those of normal lung fibroblasts for understanding the mechanisms underlying lung fibroblast-dependent antifibrotic effects of pirfenidone. In addition, we focused on the two molecular targets among previously reported 12 IPF lung-relevant translational gene markers, namely, collagen triple helix repeat containing protein 1 (CTHRC1) and four-and-a-half LIM domain protein 2 (FHL2); the levels of these two proteins were stimulated in the bleomycin-induced lung fibrosis model and attenuated by pirfenidone. Furthermore, TGF-β1–induced CTHRC1 secretion from normal primary human lung fibroblasts was inhibited by pirfenidone [16]. Meanwhile, gene expression microarray analysis revealed that both CTRHC1 and FHL2 were upregulated in IPF lung tissue compared to in control lung tissue [17, 18]. We investigated whether these can be new clinical markers for the predicting responses to pirfenidone, which will assist in selecting therapy based on in vitro functional measurements of lung fibrotic fibroblasts. Our results provided important insights into pirfenidone-mediated antifibrotic processes.

## Methods

### Materials

Cell culture medium (Dulbecco’s modified Eagle’s medium [DMEM]) was purchased from Wako (Osaka, Japan). Fetal calf serum (FCS) was purchased from Sigma-Aldrich (St. Louis, MO, USA). TGF-β1 was obtained from R & D Systems (Minneapolis, MN, USA), and rhCTHRC1 was from Abcam (Cambridge, UK). Pirfenidone was procured from Shionogi & Co. Ltd. (Osaka, Japan) and was dissolved in 100% dimethyl sulfoxide (DMSO). The amount of DMSO added did not affect the results of the bioassays [19]. Preliminary experiments with MTT demonstrated that the concentrations of pirfenidone and DMSO used in this study were not significantly cytotoxic for the fibroblasts (data not shown).

### Cell culture

Human fetal lung fibroblast-1 (HFL-1) cells were obtained from the American Type Culture Collection (CCL-153; Manassas, VA, USA). Primary lung fibroblasts were obtained from 12 patients with lung fibrosis, as diagnosed by the clinicopathological information evaluated using multidisciplinary diagnosis (MDD) team using the gold standard approach [20], and 12 patients without clinical airway symptoms or lung functional abnormalities were included in the control group (Table 1). Human primary lung parenchymal fibroblasts from patients undergoing lung resection were cultured as described previously [21]. The Institutional Review Board of Juntendo University School of Medicine and Kanagawa Cardiovascular and Respiratory Center approved the procedures. All patients provided written informed consent (approval no. 2012173).

**Table 1:** Clinical and demographic characteristics of the patients

Characteristics	Control group	PF group	*P* value
Age, y	64.5 ± 8.1	59.1 ± 14.7	0.28
Sex, (male/female)	9/3	8/4	1.00
Smoking status (yes/no)	8/4	8/4	1.00
Pack-years	712.5 ± 374.4	537.5 ± 498.2	0.44
% FVC	99.2 ± 4.1	85.2 ± 4.8	0.04
KL-6 (U/mL)	None	1422.3 ± 595.0	
SP-D (ng/mL)	None	200.7 ± 77.8	
Clinical diagnosis	None	IPF(3), NSIP(5), CHP(4)*	
Histological pattern	None	UIP(6), NSIP(6)	

Abbreviations: CHP, chronic hypersensitivity pneumonitis; FVC, forced vital capacity; KL: Krebs von den Lungen; SP: surfactant protein; PF: pulmonary fibrosis. IPF, idiopathic pulmonary fibrosis; NSIP, nonspecific interstitial pneumonia; UIP, usual interstitial pneumonia. *, Diagnosed using multidisciplinary diagnosis (MDD)

### Fibroblast chemotaxis

HFL-1 cell chemotaxis was assessed using the Boyden blind well chamber technique (Neuroprobe, Inc., Gaithersburg, MD, USA) as described previously [22]. Pirfenidone, TGF-β1, and rhCTHRC1 were added to the Swells of the upper chamber, whereas human fibronectin (20 μg/mL) was placed in the bottom chamber as the chemoattractant. The two wells of the Boyden blind well chamber were separated by an 8-μM pore filter (Nucleopore, Pleasanton, CA, USA). The chambers were incubated at 37 °C in a humid atmosphere containing 5% CO_2_ for 8 h, after which the cells on top of the filter were removed by scraping [8, 11, 23]. The cells that had migrated through the filter were then fixed, stained with DiffQuick Sysmex (16920), and mounted on glass microscope slides. Migration was assessed by counting the number of cells in five high-power fields. Wells with serum-free DMEM were used as negative controls.

### Collagen gel contraction assay

Type I collagen (rat tail tendon collagen) was extracted from rat tail tendons as described previously [24]. The effects of pirfenidone on fibroblast-mediated gel contraction were determined in the presence or absence of TGF-β1 or rhCTHRC1 using a modification of the method developed by Bell et al. [25]. The floating gels were cultured for up to 3 days, and the ability of the fibroblasts to contact the gels was determined by quantifying the area of the gels daily using an LAS4000 image analyzer (GE Healthcare Bio-Science AB, Uppsala, Sweden). Data are expressed as the gel area percentage compared to the original gel size.

### Measurement of CTHRC1, TGF-β1, and prostaglandin E2 (PGE2) levels

Cultures were maintained for 48 h to quantify CTHRC1, TGF-β1, and PGE2 levels. After 48 h, media were collected, frozen, and stored at − 80 °C until analysis. CTHRC1, TGF-β1, and PGE2 production by the cells was determined using human CTHRC1 (LifeSpan BioScienceds, Inc., Seattle, WA, USA), TGF-β1 (R&D Systems), and PGE_2_ immunoassays (Cayman Chemical, Ann Harbor, MI, USA), respectively, according to the manufacturers’ instructions.

### Western blot analysis

To standardize culture conditions, cells were passaged at a density of 5 × 10^4^ cells/mL, cultured for 48 h, and then collected for preparation of whole cell lysates. The medium was changed to DMEM without serum for 24 h, followed by treatment with TGF-β1 (0.25 ng/mL = 10pM) or rhCTHRC1 (100 ng/mL) in the presence or absence of pirfenidone (100 μg/mL) for 48 h or with various concentrations of rhCTHRC1 for 8 h. Primary antibodies against the following proteins were used for western blotting: CTHRC1 (1:5000 dilution; Proteintech, Rosemont, IL, USA; cat. no. 16534–1-AP), FHL2 (1:1000 dilution; Abcam, Cambridge, UK; cat. no. ab66399), α-SMA (1:1000 dilution; Sigma-Aldrich; cat. no. A2547), fibronectin (1:1000 dilution; Enzo Life Sciences, Inc., Farmingdale, NY, USA; cat. no. BML-FG6010–0100), bone morphogenic protein-4 (BMP-4) (1:1000 dilution; Abcam; cat. no. ab39973), Gremlin1 (1:1000 dilution; Thermo Fisher Scientific, Waltham, MA, USA; cat. no. PA5–13123), β-actin (1:5000 dilution; Wako Pure Chemical Industries; cat. no. 013–24,553), and cyclooxygenase 2 (COX2) (1:1000 dilution; Abcam; cat. no. ab169782). The bound antibodies were visualized using peroxidase-conjugated secondary antibodies and enhanced chemiluminescence using a LAS4000 image analyzer (GE Healthcare Bio-Science AB), and band intensity was analyzed Image Gauge software (LAS-400 Plus; Fujifilm).

### Immunofluorescence

HFL-1 cells were grown on Lab-Tek chamber II slides containing 10% FCS-supplemented DMEM. Following this, the cells were grown with serum-free media containing TGF-β1 (0.25 ng/mL) in the presence or absence of pirfenidone (100 μg/mL) for 48 h. HFL-1 cells were washed thrice in PBS and then fixed in 4% formaldehyde for 30 min. Permeabilization was performed in buffer containing 0.1% Triton and 0.1% sodium citrate for 5 min at 4 °C. The cells were sequentially incubated with monoclonal anti-CTHRC1 (1:50 dilution; Proteintech, Rosemont, IL, USA; cat. no. 16534–1-AP) and anti-FHL2 (1:50 dilution; Abcam, Cambridge, UK; cat. no. ab66399). The proteins were visualized by incubation with secondary antibody labeled with Alexa Fluor 488 goat anti-rabbit IgG (Invitrogen, Carlsbad, CA, USA). Images were obtained using Axioplan 2 imaging system (ZEISS, Oberkochen, Germany) with AxioVision software (ZEISS). Images used for comparisons of different cells and/or treatments were acquired using the same instrument settings and exposure times and were processed similarly.

### Small interfering RNA (siRNA)-mediated knockdown assays

Commercial siRNAs targeting *CTHRC1* (10,620,318; Life Technologies, Carlsbad, CA, USA) and *FHL2* (1,027,416; Qiagen, Valencia, CA, USA) were transfected using the RNAiMAX transfection reagent (13778–150; Life Technologies) diluted in Opti-minimal essential medium (MEM) (31,985,062; Gibco/Life Technologies) according to the manufacturers’ instructions. HLF-1 cells were plated at 1 × 10^5^ cells/mL and incubated for 24 h and were used for transfection when they were 50–70% confluent. Predetermined concentrations of siRNA were used to achieve more than 70% knockdown. To suppress endogenous *CTHRC1* and *FHL2* in fibroblasts, the cells were transfected for 24 h with 50 nM *CTHRC1* siRNA or 15 nM *FHL2* siRNA. A scrambled siRNA probe was used as a control. After silencing *CTHRC1* or *FHL2* with siRNAs, the cells were analyzed using western blotting and collagen gel contraction assays and chemotaxis experiments were performed.

### Statistical analysis

Results are expressed as means ± standard errors of the means (SEMs). Grouped data in HFL-1 cells, were evaluated using one-way analysis of variance (ANOVA) with Bonferroni correction. Samples of primary lung fibroblasts that appeared different within a series were assessed using Student’s *t*-test. For experiments in which paired samples within a group were available, we used paired Student’s *t*-tests. For these comparisons, each patient was considered an individual data point. Differences with *P* values less than 0.05 were considered significant. Data were analyzed using Prism 6 software (GraphPad Inc., San Diego, CA, USA).

## Results

### Clinical and demographic characteristics

The clinical and demographic characteristics of the patients are shown in Table 1. The pulmonary fibrosis (PF) and the control group were similar in terms of age, smoking status, and sex. However, their lung functions were significantly different; as expected, patients with lung fibrosis had lower percentage forced vital capacity (% FVC). Histological examination revealed that out of 12 patients with lung fibrosis who did not receiving medication, six had nonspecific interstitial pneumonia (NSIP), and six others had usual interstitial pneumonia (UIP). Clinical diagnoses revealed three patients with IPF, five patients with NSIP, and four patients with chronic hypersensitivity pneumonitis (CHP). Diagnosed using multidisciplinary diagnosis (MDD) according to the American Thoracic Society/European Respiratory Society (ATS/ERS) International Multidisciplinary Consensus Classification of the Idiopathic Interstitial Pneumonias guidelines [26].

### Effects of pirfenidone on TGF-β1-stimulated fibroblast activity

**Fig. 1:**
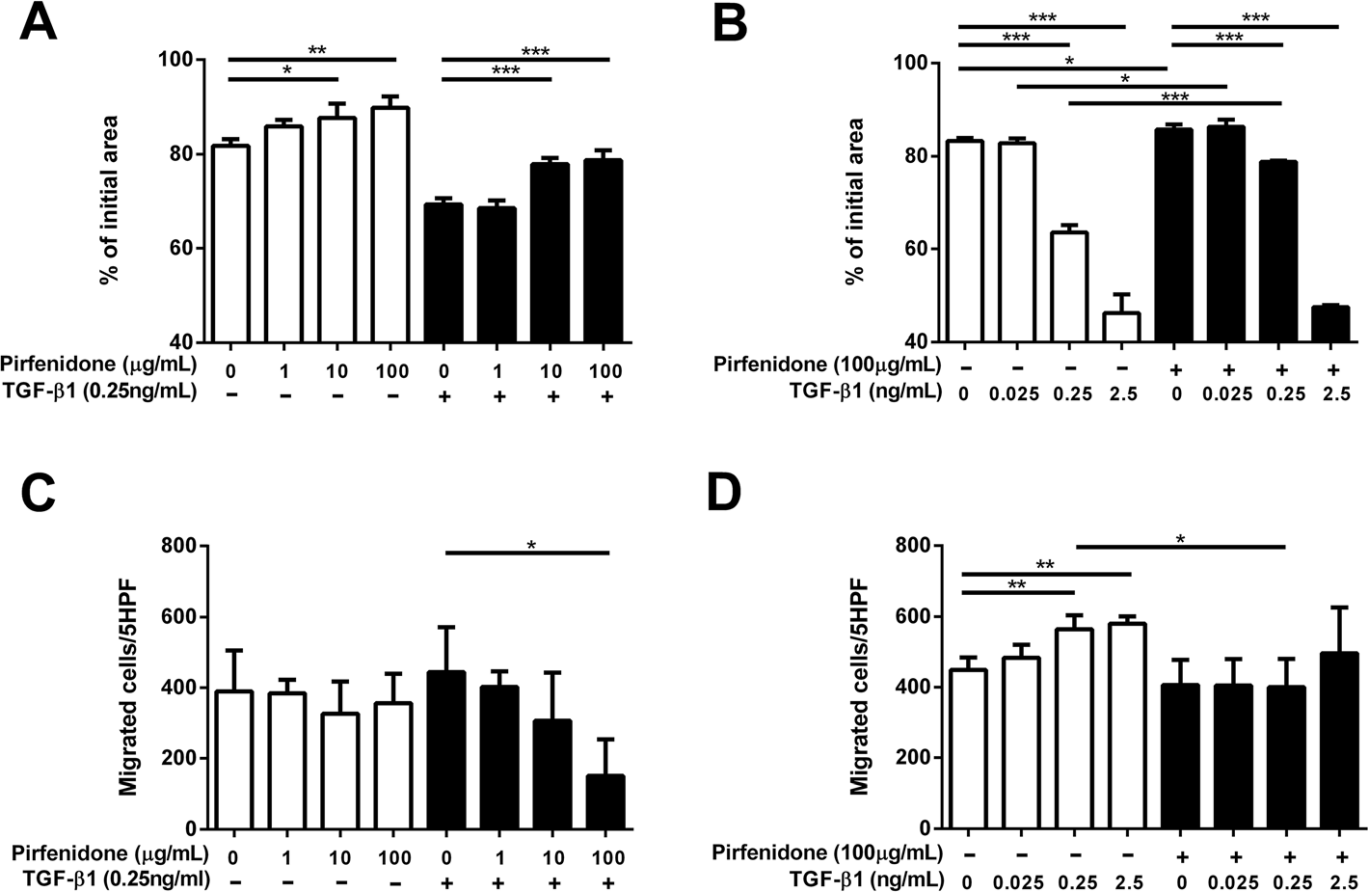
Effects of pirfenidone on TGF-β1-stimulated collagen gel contraction and chemotaxis in HFL-1 cells. HFL-1 cells were cultured and cast into three-dimensional collagen gels that were maintained in suspension; the gel size was measured daily. HFL-1 cells grown in a monolayer culture were trypsinized and their chemotactic activity toward fibronectin (20 μg/mL) was assessed using the Boyden blind well chamber technique. (**a**) Collagen gel contraction in the presence of various concentrations of pirfenidone in the presence or absence of 0.25 ng/mL (10 pM) TGF-β1. (**b**) Collagen gel contraction following treatment with various concentrations of TGF-β1 in the presence or absence of 100 μg/mL pirfenidone. (**c**) The number of migrated fibroblasts following treatment with various concentrations of pirfenidone in the presence or absence of 0.25 ng/mL TGF-β1. (**d**) Number of migrated fibroblasts following treatment with various concentrations of TGF-β1 in the presence or absence of 100 μg/mL pirfenidone. Collagen gel contraction vertical axis: gel size measured after 2 days of contraction expressed as a percentage of the initial value. Chemotaxis vertical axis: number of migrated cells per five high-power fields (5 HPF). Horizontal axis: conditions. Values represent means ± SEMs of three to five separate experiments, each of which included three replicates. *P < 0.05, **P < 0.01, ***P < 0.001

**Fig. 2:**
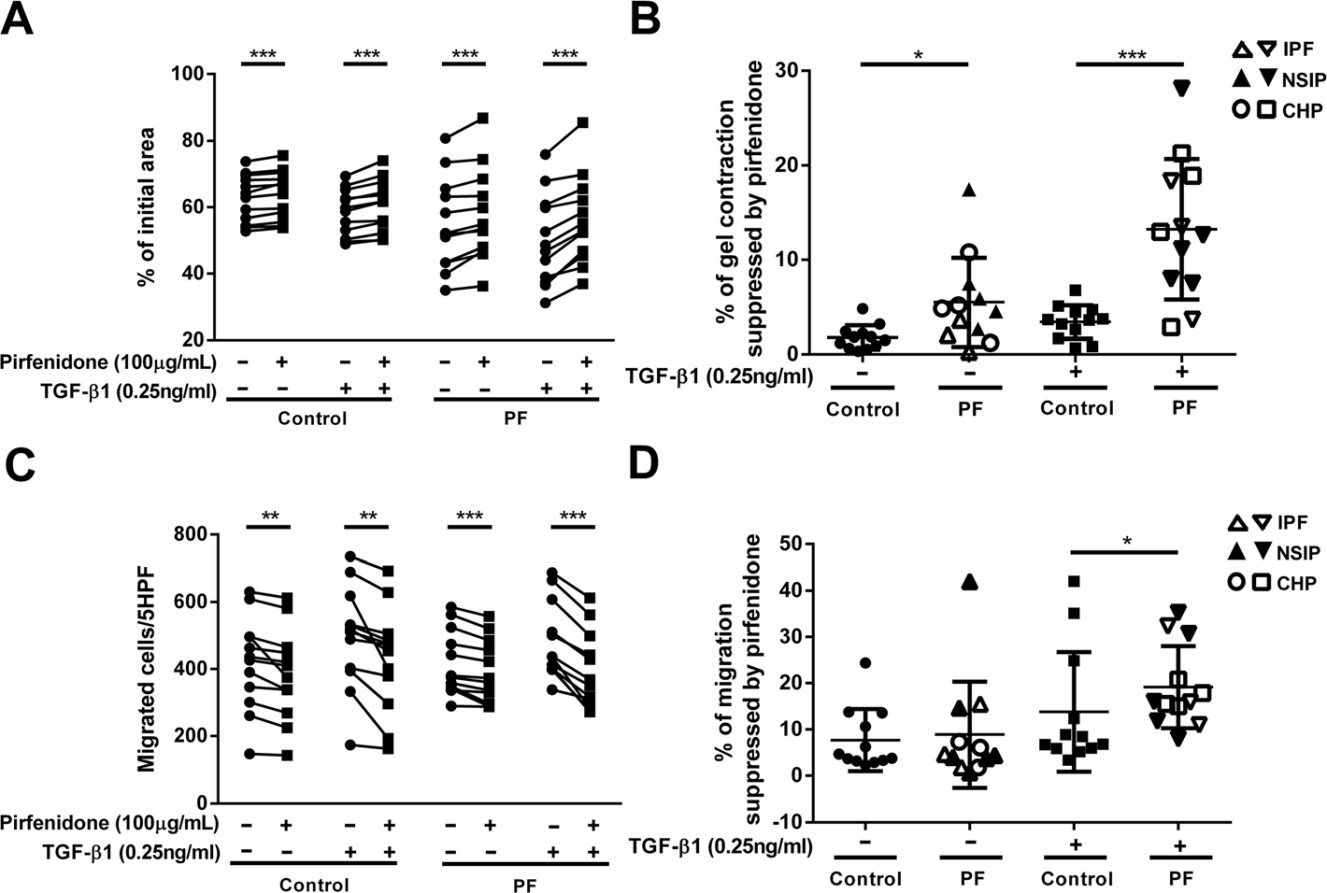
Effects of pirfenidone on collagen gel contraction and chemotaxis in primary lung fibroblasts. Fibroblasts from controls and patients with pulmonary fibrosis (PF) were cultured in the presence or absence of pirfenidone (100 μg/mL) and 0.25 ng/mL TGF-β1, and collagen gel contraction and chemotaxis were assayed. (**a**) Collagen gel contraction. Vertical axis: gel size measured after 2 days of contraction expressed as a percentage of the initial gel area. Horizontal axis: conditions. (**b**) Pirfenidone-induced suppression of collagen gel contraction in lung fibroblasts isolated from controls and patients with IP. Vertical axis: percentage of gel contracted size following pirfenidone treatment ([difference in gel size/initial gel size] × 100). Horizontal axis: conditions. (**c**) Fibroblast chemotaxis. Vertical axis: number of migrated fibroblasts per 5 HPF. Horizontal axis: conditions. (**d**) Pirfenidone-dependent suppression of chemotaxis in lung fibroblasts isolated from controls and patients with IP. Vertical axis: percentage of migrated cells following pirfenidone treatment ([difference in migrated cell number/initial migrated cells number] × 100). Horizontal axis: conditions. Each patient evaluated was expressed as an individual symbol, representing the means of two to three separate experiments, each of which included three replicates. (**a**, **c**) Lines connect the values for individual patients in the presence or absence of pirfenidone. *P < 0.05, **P < 0.01, ***P < 0.001. (**b**, **d**) Student’s paired t test and unpaired t test were used. *P < 0.05, **P < 0.01, ***P < 0.001

Pirfenidone inhibited collagen gel contraction of HFL-1 cells in a concentration-dependent manner, but did not affect chemotaxis when added alone to these cells. Next, we investigated whether pirfenidone altered the TGF-β1-induced increase in collagen gel contraction and chemotaxis towards fibronectin in HFL-1 cells. Pirfenidone treatment reduced TGF-β1-induced collagen gel contraction and chemotaxis in HFL-1 cells in a concentration-dependent manner (*P* < 0.05 for 100 μg/mL pirfenidone ±0.25 ng/mL (10pM) TGF-β1 versus control; Fig. 1A, C). Pirfenidone abolished gel contraction and chemotaxis in the presence of less than 0.25 ng/mL TGF-β1 (*P* < 0.05). However, in the presence of 2.5 ng/mL (100pM) TGF-β1, pirfenidone did not inhibit gel contraction and chemotaxis of HFL-1 cells (Fig. 1B, D). Although the maximum plasma concentration (*C*_max_) of pirfenidone, which is typically used for treating patients with IPF at dosage up to 1800 mg/day, is 15.7 μg/mL after administration of 801 mg pirfenidone [27], higher concentrations may be used; indeed, pirfenidone is widely used at concentrations above 100 μg/mL in vitro in the laboratory setting [28]. Therefore, we used 100 μg/mL pirfenidone, with or without 0.25 ng/mL (10pM) TGF-β1, in our subsequent experiments on adult human primary lung fibroblasts. Notably, gel contraction and chemotaxis were attenuated in cells treated with 100 μg/mL pirfenidone alone or in combination with 0.25 ng/mL TGF-β1 (Fig. 2A, C, *P* < 0.05). This inhibitory effect was higher in fibroblasts from fibrotic lungs than in control fibroblasts, especially with TGF-β1 treatment (fibrotic vs. normal lung, with or without TGF-β1 treatment; gel contraction: *P* < 0.001 and *P* < 0.05, chemotaxis: *P* < 0.05 and *P* = 0.16; Fig. 2B, D). However, there were no differences in the effects among the fibrotic fibroblasts from patients with clinical diagnosis of IFP, NSIP and CHP. At the end of the incubation, cell numbers or viability in the gels in pirfenidone- and/or TGF-β1-treated groups were not different from those in the control group, as assessed using 3-(4,5-dimethylthiazol-2-yl)-2,5-diphenyltetrazolium bromide (MTT) assays (data not shown).

### High sensitivity of TGF-β1-induced fibrotic mediators in fibrotic lung fibroblasts

**Fig. 3:**
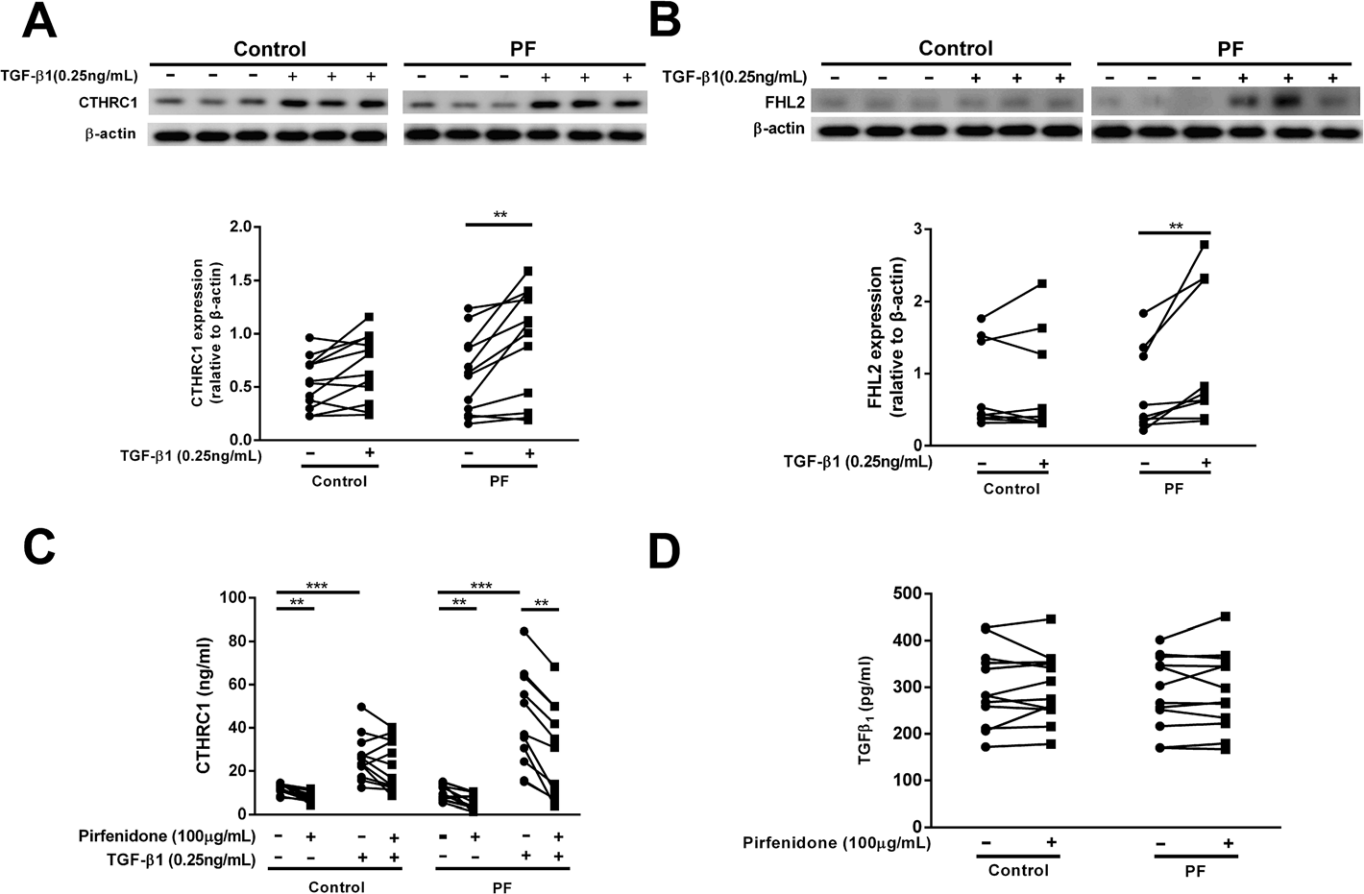
High sensitivity of TGF-β1-induced fibrotic mediators in fibrotic lung fibroblasts. Subconfluent fibroblasts from 12 controls and 12 patients with PF were cultured in serum-free (SF)-DMEM for 24 h and then incubated in the presence or absence of TGF-β1 (0.25 ng/mL) and/or pirfenidone (100 ng/mL) for 48 h. Proteins from monolayer cultured fibroblasts were extracted and subjected to western blot analysis, and media were harvested from monolayer cultures and evaluated for CTHRC1 and TGF-β1 levels using immunoassay. Expression of (**a**) CTHRC1 (30 kDa) and (**b**) FHL2 (30 kDa) from fibroblasts isolated from controls and patients with PF in the presence or absence of TGF-β1 (0.25 ng/mL). Vertical axis: Protein expression normalized to β-actin expression. Immunoassay of (**c**) CTHRC1 and (**d**) TGF-β1. Vertical axis: mediator production expressed as an amount. Symbols represent the mean values for individual patients, as assessed in two separate experiments. Horizontal axis: conditions. Student’s paired t test was used. *P < 0.05, **P < 0.01, ***P < 0.001

CTHRC1 is a marker of activated stromal cells [29]. As pirfenidone suppresses both TGF-β1-induced CTHRC1 and FHL2 [16], we assessed TGF-β1-induced changes in CTHRC1 and FHL2 expression levels in fibrotic fibroblasts using immunoblotting. The relative increases in CTHRC1 and FHL2 levels upon TGF-β1 stimulation were higher in fibrotic fibroblasts than in control fibroblasts. However, CTHRC1 and FHL2 levels in the normal control fibroblast did not change upon TGF-β1 stimulation (CTHRC1: control, *P* = 0.109 versus lung fibrosis, *P* = 0.003; FHL2: control, *P* = 0.360 versus lung fibrosis, *P* = 0.006; Fig. 3A, B), which suggested that fibrotic fibroblasts specifically responded to TGF-β1 stimulation as previously described [11]. As CTHRC1 is a secreted protein, and a previous report showed that CTHRC1 was present in patient plasma [29], we measured release of CTHRC1 from human lung fibroblasts using enzyme-linked immunosorbent assay (ELISA). The relative increase in the amount of CTHRC1 released upon TGF-β1 stimulation was higher from fibrotic fibroblasts than from control fibroblasts. The fold increase in CTHRC1 in the presence of TGF-β1 stimulation (1.347 ± 0.246 in control fibroblasts versus 3.610 ± 0.662 in fibrotic fibroblasts; *P* = 0.004) was higher in fibroblasts from fibrotic lungs than in control fibroblasts. The suppressive effects of pirfenidone on CTHRC1 release from TGF-β1-induced fibroblasts was higher in fibrotic lung fibroblasts than in control fibroblasts (control: *P* = 0.111; IP: *P* < 0.001); in contrast, the suppressive effects of pirfenidone alone were similar with respect to release of CTHRC1 (control: *P* = 0.004; IP: *P* = 0.001). The fold reduction in CTHRC1 in the presence of pirfenidone following TGF-β1 stimulation (0.139 ± 0.069 in control fibroblasts versus 0.400 ± 0.064 in fibrotic fibroblasts; *P* = 0.018) was higher in fibroblasts from fibrotic lungs than in control fibroblasts (all fold changes were calculated as difference in value after treatment / initial value) (Fig. 3C).

Fibroblasts are known to release mediators, including TGF-β1 [7, 8] and PGE_2_ [30, 31], which can modulate chemotaxis and collagen gel contraction in an autocrine or paracrine manner. To determine whether these mediators directly contribute to the pirfenidone-mediated suppression of collagen gel contraction and chemotaxis, the release of these mediators in the monolayer culture medium was evaluated using ELISA. Notably, pirfenidone did not affect TGF-β1 levels in culture medium of control and fibrotic fibroblasts (Fig. 3D). Furthermore, the ability of pirfenidone to modulate PGE_2_ release and inducible cyclooxygenase 2 (COX2) expression was further assessed in the culture medium of HFL-1 cells. Pirfenidone did not stimulate PGE_2_ release or COX2 expression with or without TGF-β1 in fibroblasts (Additional file 1: Figure S1 A, B). These results indicated that CTHRC1 and FHL2 play critical roles in pirfenidone-mediated regulation of fibroblast activity and do not exert direct effects on TGF-β1-mediated autocrine/paracrine regulation in fibroblasts.

### Effects of pirfenidone on TGF-β1-mediated fibrotic regulators in lung fibroblasts

**Fig. 4:**
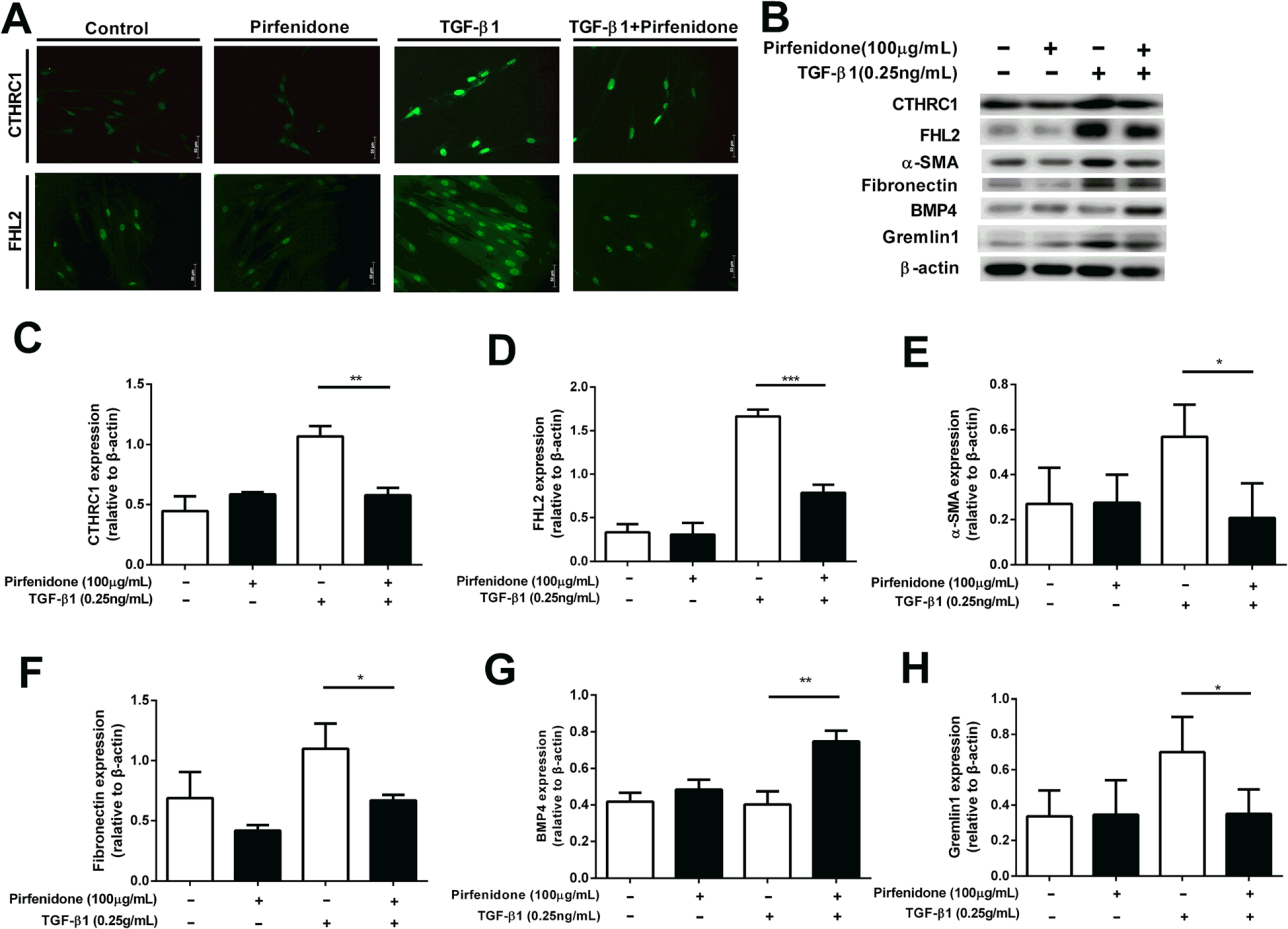
Effects of pirfenidone on TGF-β1-mediated fibrotic regulators in lung fibroblasts. Subconfluent HFL-1 cells were cultured in SF-DMEM for 24 h and then incubated in the presence or absence of TGF-β1 (0.25 ng/mL) and pirfenidone (100 ng/mL). (**a**) CTHRC1 and FHL2 expression in HFL-1 cells was determined using fluorescence-immunohistochemistry with primary antibodies against CTHRC1 or FHL2, followed by incubation with secondary antibodies labeled with Alexa Fluor 488 goat anti-rabbit IgG (green). The scale bar indicates 50 μm. (**b**) Western blot analysis to determine the effects of pirfenidone on targets related to TGF-β1-mediated fibrotic processes, including CTHRC1 (30 kDa), FHL2 (30 kDa), α-SMA (42 kDa), fibronectin (250 kDa), BMP4 (47 kDa), Gremlin1 (25 kDa), and β-actin (42 kDa). The vertical axis shows the relative intensities of (**c**) CTHRC1, (**d**) FHL2, (**e**) α-SMA, (**f**) fibronectin, (**g**) BMP4, and (**h**) Gremlin1 versus β-actin; the horizontal axis shows the conditions. Values represent means ± SEMs of three to five separate experiments. *P < 0.05, **P < 0.01, ***P < 0.001

Next, we assessed whether pirfenidone altered the expression of targets related to TGF-β1-mediated fibrotic processes in HFL-1 cells. As CTHRC1 and FHL2 are located both in the cytoplasm and nucleus [32, 33], we evaluated the effects of pirfenidone on the localization of the TGF-β1-induced fibrosis regulators using immunofluorescence analysis. TGF-β1 strongly enhanced both CTHRC1 and FHL2 staining, whereas pirfenidone suppressed TGF-β1-induced CTHRC1 and FHL2 expression, including the nuclear localization (fluorescein isothiocyanate stain; green immunofluorescence: Fig. 4A). Western blot analysis showed that treatment with pirfenidone significantly reduced TGF-β1-augmented expression of CTHRC1, FHL2, α-SMA, fibronectin, and Gremlin1 (*P* < 0.05; Fig. 4B–F, H) and reversed TGF-β1-dependent suppression of BMP4 (*P* < 0.05; Fig. 4B, G).

### Effects of pirfenidone on CTHRC1-mediated regulation in lung fibroblasts

**Fig. 5:**
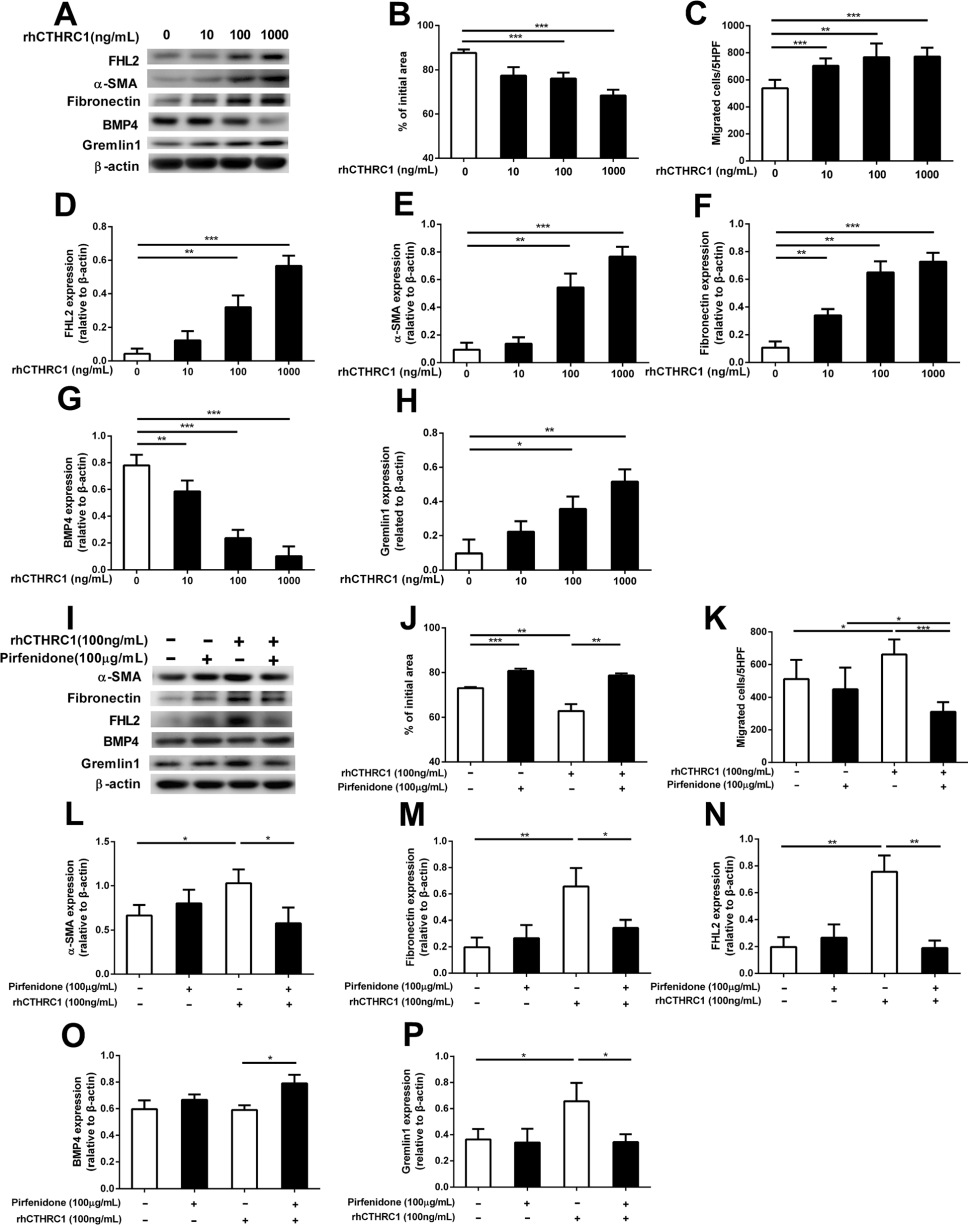
Effects of pirfenidone on CTHRC1-mediated regulation in lung fibroblasts. Subconfluent HFL-1 cells were cultured in SF-DMEM for 24 h and then incubated in the presence or absence of different concentrations of rhCTHRC1. Western blot analysis of the effects of different concentrations of rhCTHRC1 on targets related to fibrotic processes (**a**). Effects of different concentrations of rhCTHRC1 on HFL-1 cell-mediated collagen gel contraction (**b**) and chemotaxis (**c**). Effects of different concentrations of rhCTHRC1-mediated targets assayed using western blot analysis (**d**-**h**). The vertical axis shows the relative intensities of FHL2 (D), α-SMA (**e**), fibronectin (**f**), BMP4 (**g**), Gremlin1 (**h**) versus β-actin; the horizontal axis shows the conditions. Subconfluent HFL-1 cells were cultured in SF-DMEM for 24 h and then incubated in the presence or absence of rhCTHRC1 (100 ng/mL) and pirfenidone (100 ng/mL) for 48 h. Western blot analysis to analyze the effects of pirfenidone on rhCTHRC1-mediated targets related to fibrotic processes (**i**). Effects of pirfenidone on rhCTHRC1-mediated collagen gel contraction (**j**) and chemotaxis (**k**). Effects of pirfenidone on the expression levels of rhCTHRC1-mediated targets assayed using western blot analysis (**l**-**p**). The vertical axis shows the relative intensities of α-SMA (**l**), fibronectin (**m**), FHL2 (**n**), BMP4 (**o**), Gremlin1 (**p**) versus β-actin; the horizontal axis shows the conditions. Collagen gel contraction, vertical axis: gel size measured after 2 days of contraction expressed as a percentage of the initial value. Chemotaxis, vertical axis: number of migrated cells per 5 HPF. Horizontal axis: conditions. Values represent means ± SEMs of three to five separate experiments. *P < 0.05, **P < 0.01, ***P < 0.001

We investigated the effects of recombinant human (rh) CTHRC1 on HFL1-mediated collagen gel contraction and chemotaxis. Compared to the control, rhCTHRC1 (10–1000 ng/mL) stimulated gel contraction and chemotaxis toward fibronectin in a concentration-dependent manner (*P* < 0.05) (Fig. 5A–C), accompanied by concentration-dependent upregulation of FHL2, α-SMA, fibronectin, and Gremlin1 (Fig. 5A, D–F, H) and downregulation of BMP4 (Fig. 5A, G). According to a previous report on detection of CTHRC1 plasma levels, we used 100 ng/mL rhCTHRC1 with or without 100 μg/mL pirfenidone in our studies on HFL-1 cells [29]. Pirfenidone significantly attenuated rhCTHRC1-induced gel contraction and chemotaxis (Fig. 5J, K); it also suppressed rhCTHRC1-induced increase in FHL2, α-SMA, fibronectin, and Gremlin1 expression (Fig. 5I, L-N, P) and reversed rhCTHRC1-dependent suppression of BMP4 (Fig. 5I, O).

### Inhibition of CTHRC1- and FHL2-mediated regulation of HFL-1

**Fig. 6:**
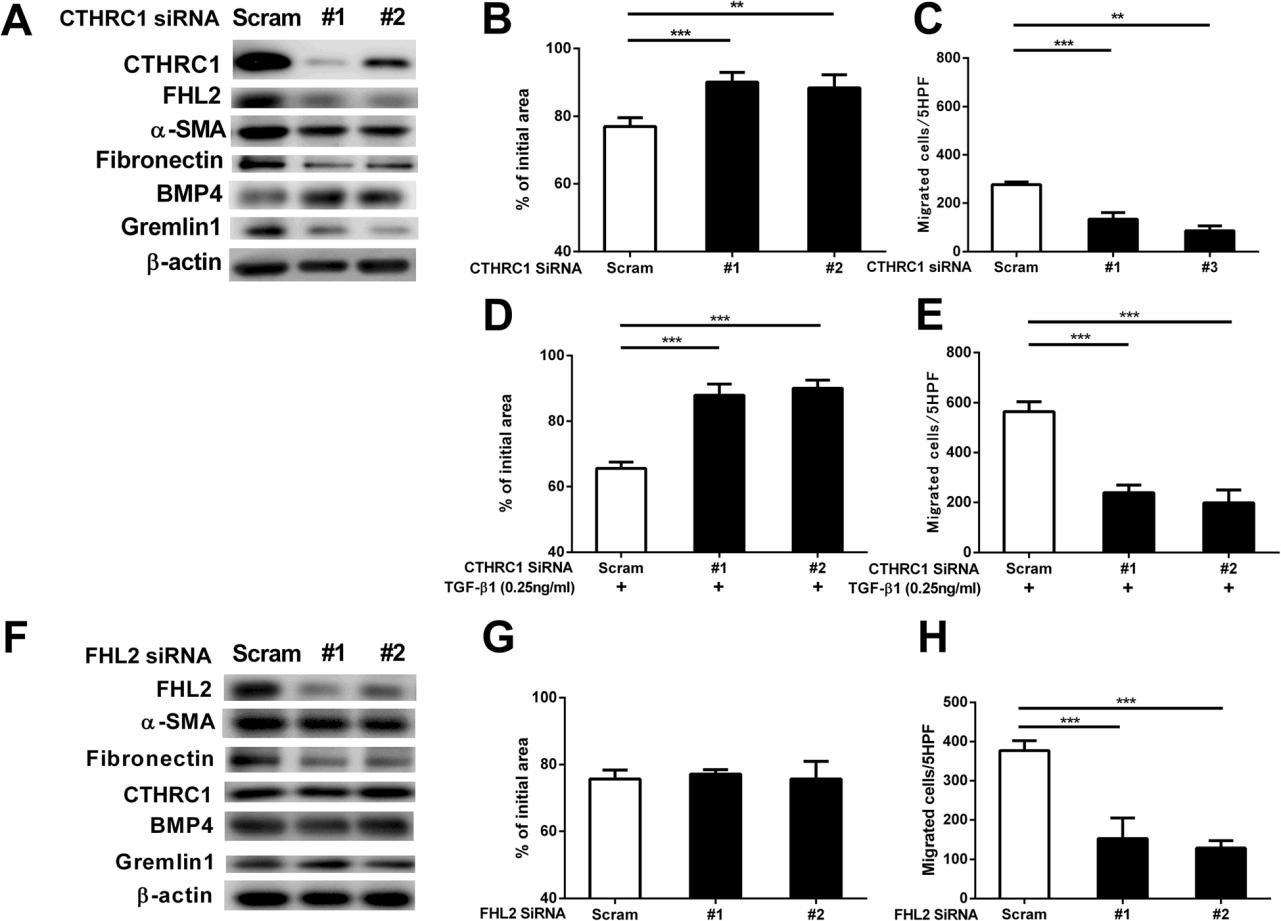
Effects of *CTHRC1* and *FHL2* knockdown in HFL-1 cells. Collagen gel contraction and chemotaxis were assessed in *CTHRC1*- and *FHL2*-knocked down HFL-1 cells. Western blot analysis of the effects of *CTHRC1* silencing on targets related to fibrotic processes (**a**). Collagen gel contraction (**b**) and chemotaxis (**c**) after silencing of *CTHRC1*. The effect of *CTHRC1* knockdown on TGF-β1 (0.25 ng/mL)-induced gel contraction (**d**) and chemotaxis toward to fibronectin (**e**). Western blot analysis to analyze the effects of *FHL2* silencing on targets related to fibrotic processes (**f**). Collagen gel contraction and (**g**) chemotaxis (**h**) after silencing of *FHL2*. Collagen gel contraction, vertical axis: gel size measured after 2 days of contraction expressed as a percentage of the initial value. Chemotaxis, vertical axis: number of migrated cells per 5 HPF. Horizontal axis: conditions. Values represent means ± SEMs of three separate experiments. *P < 0.05, **P < 0.01, ***P < 0.001

To investigate the roles of the pirfenidone- targets CTHRC1 and FHL2 in lung fibroblasts, we knocked down these genes in HFL-1 cells. Silencing of *CTHRC1* (Fig. 6A, E2A) reversed the TGF-β1-mediated fibrotic processes, i.e., reduction in FHL2 (Additional file 2: Figure S2 B), α-SMA (Additional file 2: Figure S2 C), fibronectin (Additional file 2: Figure S2 D), and Gremlin1 (Additional file 2: Figure S2 F) expression and increase in BMP4 expression (Additional file 2: Figure S2 E). Furthermore, *CTHRC1* knockdown attenuated gel contraction and chemotaxis toward to fibronectin (Fig. 6B, C). Since, CTHRC1 released upon TGF-β1 stimulation was higher from fibrotic fibroblasts, we investigated the effect of *CTHRC1* knockdown on TGF-β1-induced gel contraction and chemotaxis toward fibronectin. *CTHRC1* knockdown further attenuated gel contraction and chemotaxis in the presence of TGF-β1 (Fig. 6D, E)**.** However, silencing of *FHL2* (Fig. 6F, Additional file 2: Figure S2 G) did not affect CTHRC1 (Additional file 2: Figure S2 H), α-SMA (Additional file 2: Figure S2 I), BMP4 (Additional file 2: Figure S2 K), and Gremlin1 (Additional file 2: Figure S2 L) expression and gel contraction (Fig. 6G), but reduced fibronectin expression (Additional file 2: Figure S2 J) and chemotaxis toward to fibronectin (Fig. 6H).

### Effects of pirfenidone on in vitro TGF-β1-stimulated fibroblast activity and biomarkers of lung fibrosis

**Fig. 7:**
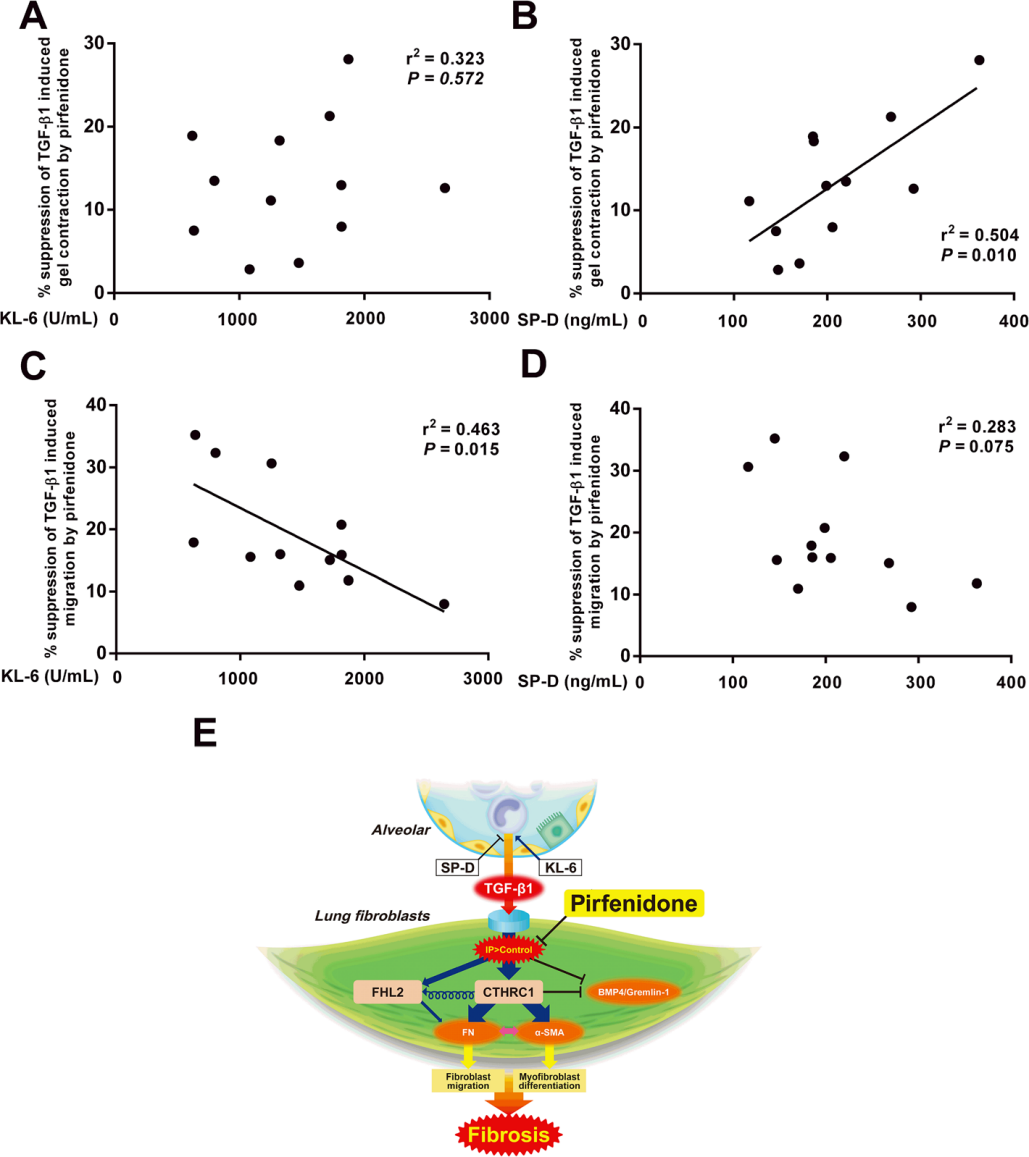
Relationship between pirfenidone’s effect on TGF-β1-stimulated fibroblast bioactivity in vitro and biomarkers of lung fibrosis. Comparison of the relationships between suppression of TGF-β1-induced gel contraction by pirfenidone and serum (**a**) KL-6 and (**b**) SP-D levels, and between suppression of TGF-β1-induced migration and (**c**) KL-6 and (**d**) SP-D levels. Symbols represent individual patients. Linear regression was used. *P* < 0.05 indicates a positive relationship between pirfenidone response to TGF-β1-stimulated fibroblast bioactivity in vitro and biomarkers of lung fibrosis. (**e**) Schematic showing the mechanism via which pirfenidone controls TGF-β1-induced changes in fibrotic lung fibroblasts. Lung fibroblasts are continually exposed to TGF-β1 via airway cells-fibroblasts interaction, which is regulated by mediators released by airway cells under inflammatory conditions, resulting in distinct phenotype of fibrotic fibroblasts. This high sensitivity to TGF-β1, along with upregulation of CTHRC1 and FHL2, leads to the development of fibrosis in fibrotic fibroblasts. Treatment with pirfenidone can effectively block this process

Considering our observation that pirfenidone increased the sensitivity of fibrotic lung fibroblasts to TGF-β1-induced fibrosis compared to normal fibroblasts, we investigated whether our in vitro data was clinically significant. Toward this objective, we measured serum levels of the lung fibrosis biomarkers KL-6 and SP-D in the serum of patients with lung fibrosis at the time of primary lung fibroblast sample collection. The ability of pirfenidone to abrogate a TGF-β1-induced increase in collagen gel contraction correlated positively with SP-D levels (r^2^ = 0.504, *P* = 0.010, Fig. 7B) but not with KL-6 levels (Fig. 7A). In contrast, the inhibitory effects of pirfenidone on TGF-β1-induced migration correlated negatively with KL-6 levels (r^2^ = 0.463, *P* = 0.015, Fig. 7C) but not with SP-D levels (Fig. 7D). No other clinical or histopathological and spirometric parameters, such as sustainer and rapid decliner categories according to forced vital capacity (FVC) reduction rates, were related to in vitro fibroblast response to pirfenidone treatment or to in vitro fibroblast activity.

## Discussion

In this study, we observed that pirfenidone suppressed fibrotic changes in the fibrotic lung fibroblast phenotype such as collagen gel contraction and migration following TGF-β1-mediated stimulation. Previous reports have shown that pirfenidone reduces proliferation, migration, fibroblast-embedded collagen gel contraction, ECM production, and TGF-β1-mediated differentiation into myofibroblasts by attenuating the effect of TGF-β1 and its downstream targets, including phosphorylation of Smad3, connective tissue growth factor, p38, and Akt in human fibroblasts [28, 34–38]. These support our current results. Pirfenidone prevented changes in the fibrotic fibroblast phenotype such as increase in proliferation and migration and elevation in the levels of phospho-Smad3, phospho-signal transducer and activator of transcription 3 (STAT3), α-SMA, and collagen in the context of IPF [39]. We clarified the mechanisms via which TGF-β1 stimulated CTHRC1 and FHL-2 [16] in lung fibrotic fibroblasts but not in control fibroblasts. Our results showed that pirfenidone suppressed CTHRC1-induced lung fibroblasts migration toward fibronectin, gel contraction, and α-SMA and fibronectin expression, and increased the BMP4/Gremlin1 ratio. In addition, pirfenidone also suppressed FHL-2-mediated fibroblast migration. Furthermore, we previously demonstrated that the high response of fibrotic fibroblasts to TGF-β1 [11] suggested resulting in the high response of TGF-β1–induced CTHRC1 and FHL2 expression, which in turn elicited elevated response to pirfenidone treatment in fibrotic fibroblasts.

CTHRC1 is expressed by activated stromal cells of diverse origin and is co-expressed with α-SMA. Elevated CTHRC1 levels were detected in patients with inflammatory conditions, including rheumatoid arthritis, and CTHRC1 is considered a marker of tissue remodeling, inflammation, or wounding [29, 40]. A *previous report has shown that* CTHRC1 plays a protective role in pulmonary fibrosis and tissue repair and may be clinically applied for treating fibrosis as it decreases collagen matrix deposition by inhibiting Smad2/3 activation [41]. In addition, rhCTHRC1 inhibits TGF-β1-stimulated collagen type I synthesis and promotes skin repair in keloid fibroblasts [42, 43]. The systemic loss of CTHRC1 increased TGF-β1*-mediated* excess matrix deposition and induced the development of bleomycin-induced lung fibrosis in mice [44]. Furthermore, TGF-β1 and BMP4, which belong to the TGF-β superfamily of ligands, stimulate CTHRC1 expression, and overexpression of CTHRC1 in smooth muscle cells increases migration, as is also observed in embryonic fibroblasts treated with exogenous rhCTHRC1 [40, 43]. In this study, fibrotic fibroblasts showed TGF-β1-stimulated CTHRC1 expression and pirfenidone-dependent suppression of CTHRC1 expression after TGF-β1 treatment. In addition, rhCTHRC1 stimulated lung fibroblast-mediated gel contraction and migration toward fibronectin, and *CTHRC1* knockdown functionally suppressed lung fibroblasts-mediated migration and gel contraction and blocked the TGF-β1 stimulation. Pirfenidone restored TGF-β1- and rhCTHRC1-dependent suppression of BMP4 expression, which was previously reported to reduce proliferation and TGF-β1-induced migration toward fibronectin and synthesis of ECM components, including fibronectin, in lung fibroblast [11, 45, 46]. In the present study, pirfenidone also reduced the levels of the BMP4 antagonist Gremlin1, which was previously implicated in the development of lung fibrosis [47], when stimulated by TGF-β1 and rhCTHRC1. These results indicated that CTHRC1 acted as a co-stimulator of TGF-β1-mediated fibrotic processes in fibrotic fibroblasts rather than as a suppresser of fibrosis. Thus, CTHRC1 could be a dominant target for pirfenidone-mediated antifibrotic mechanisms in fibrotic lung fibroblasts.

FHL2 participates in tissue wound healing and is associated with fibrogenesis [48, 49]. *Fhl2* ^−/−^ mice develop hepatic fibrogenesis [50], and *Fhl2*-deficient embryonic fibroblasts show reduced collagen contraction and cell migration, resulting in impaired wound healing [48]. However, in contrast to CTHRC1, FHL2 has been reported to positively regulate the expression of collagen types I and III in an FHL2-induced BLM-treated lung fibrosis model; this process tended to involve acceleration of lung inflammation rather than direct FHL2-induced fibrotic mechanisms [51–53]. Furthermore, FHL2 induces α-SMA [48] and is stimulated by TGF-β1 [16, 54]. In this study, we also demonstrated that compared to control fibroblasts, FHL2 was further stimulated by TGF-β1 in fibrotic fibroblasts, and that pirfenidone suppressed TGF-β1-stimulated FHL2 expression. However, *FHL2* knockdown suppressed only fibronectin expression and migration toward fibronectin, but did not affect gel contraction, expression of other TGF-β1-mediated fibroblast regulators, and CTHRC1 expression. As only *CTHRC1* knockdown attenuated FHL2 expression, CTHRC1 may regulate FHL2 in one direction. Thus, FHL2 may be partially involved in lung fibroblast-mediated fibrosis following TGF-β1-induced upregulation of CTHRC1 (Fig. 7E).

Clinically, patients with IPF who showed predicted vital capacity (VC) of more than 70% and lowest oxygen saturation in blood during 6 min walking tests (less than 90% at baseline) are most likely to benefit from pirfenidone therapy [55]. The expression levels of pirfenidone-targeted translational gene markers (*GREM1*, *CTHRC1*, and *FHL2*) showed significant negative correlation with the percentage diffusing capacity of carbon monoxide (%DLCO) and was associated with IPF disease severity [16]. However, little is known regarding the surrogate markers of pirfenidone response. In this study, we demonstrated that the magnitude of pirfenidone-dependent suppression of TGF-β1-induced gel contraction and migration was positively related to serum SP-D levels and negatively related to serum KL-6 levels respectively, but was not related to any other clinical parameters, including histological pattern and lung function (%VC, %FVC, and %DLCO). Previous reports have demonstrated that SP-D acts as a suppressor of lung fibrosis by inhibiting TGF-β1 release from macrophages [2]; in contrast, KL-6 promoted human lung fibroblast chemotaxis and increased TGF-β1 and fibronectin release [3, 56]. Previously, we have also revealed that higher SP-D and lower KL-6 levels were associated with increased sensitivity to ERK5 inhibitor, which suppresses TGF-β1-stimulated lung fibroblast bioactivity ex vivo [11]. Thus, in the present study, we associated SP-D and KL-6 with pirfenidone response and speculated that serum SP-D and KL-6 can indicate response to pirfenidone via epithelial-mesenchymal interactions. These diverse phenotypic responses to pirfenidone in fibrotic lung fibroblasts and our in vitro results related to serum biomarkers suggested that progression of lung fibrosis with increasing SP-D/KL-6 ratios may be associated with better clinical outcomes (Fig. 7E). This analysis described the relative usefulness of other clinical parameters at baseline when determining the predictable surrogate markers of patients with lung fibrosis as candidates for pirfenidone therapy.

## Conclusions

The results presented here provided evidence regarding the high sensitivity of fibrotic fibroblasts to pirfenidone, which regulated TGF-β1-induced fibrotic processes. However, our study has certain limitations. For example, we used limited number of patient fibroblast lines and did not assess plasma levels of CTHRC1 as a clinical surrogate marker for predicting pirfenidone response. Instead, we performed functional experiments in lung fibroblasts and analyzed the responses of fibroblasts derived from patients with lung fibrosis to pirfenidone for determining the utility of CTHRC1 as a potential therapeutic target and predicting pirfenidone responses in lung fibrosis.

## Additional files

Additional file 1: **Figure S1.** Effects of pirfenidone on TGF-β1-mediated regulators of the COX2)/ PGE2 pathway in fibroblasts. Subconfluent HFL-1 cells were cultured in SF-DMEM for 24 h and then incubated in the presence or absence of TGF-β1 (10 pM) and pirfenidone (100 ng/mL) for 48 h. Proteins were extracted and subjected to western blot analysis of COX2. Media were harvested from monolayer culture and evaluated for PGE2 by immunoassay. Release of PGE2 (A) in HFL-1 monolayer cultures and western blot analysis of COX2 (69 kDa) (B). Bar figure vertical axis: protein expression relative to β-actin. Horizontal axis: conditions. Values represent means ± SEMs of at least three independent experiments. (TIF 579 kb)

Additional file 2: **Figure S2.** Effects of *CTHRC1* and *FHL2* knockdown on targets related to fibrotic processes in fibroblasts. Western blot analysis of targets related to fibrotic processes were assessed in *CTHRC1*-knocked down HFL-1 cells (A-F). The vertical axis shows the relative intensities of CTHRC1 (A), FHL2 (B), α-SMA (C), fibronectin (D), BMP4 (E), Gremlin1 (F) versus β-actin; the horizontal axis shows the conditions. Western blot analysis of targets related to fibrotic processes were assessed in *FHL2*-knocked down HFL-1 cells (G-L). The vertical axis shows the relative intensities of FHL2 (G), CTHRC1 (H), α-SMA (I), fibronectin (J), BMP4 (K), Gremlin1 (L) versus β-actin; the horizontal axis shows the conditions. Values represent means ± SEMs of three separate experiments. *P < 0.05, **P < 0.01, ***P < 0.001. (TIF 627 kb)

## Data Availability

The dataset supporting the conclusions of this article is included within the article.

## Abbreviations

%DLCO: Percent diffusing capacity of carbon monoxide
%FVC: Percent forced vital capacity
BMP-4: Bone morphogenetic protein-4
CHP: Chronic hypersensitivity pneumonitis
COX2: Cyclooxygenase 2
CTHRC1: Collagen triple helix repeat containing protein 1
ECM: Extracellular matrix
ELISA: Enzyme-linked immunosorbent assay
FHL2: Four-and-a-half LIM domain protein 2
HFL-1: Human fetal lung fibroblast-1
IPF: Idiopathic pulmonary fibrosis
KL: Krebs von den Lungen
MDD: Multidisciplinary diagnosis
NSIP: Nonspecific interstitial pneumonia
PDGF: Platelet-derived growth factor
PF: Pulmonary fibrosis
PGE2: Prostaglandin E2
Rh: Recombinant human
SEM: Standard errors of the means
SP: Surfactant protein
TGF: Transforming growth factor
UIP: Usual interstitial pneumonia
VC: Vital capacity
α-SMA: α-smooth muscle actin
